# Feeding All-Trans Retinoic Acid to Pregnant Sows Regulates the Development of the Pulmonary Nervous Systems of Neonatal Pigs

**DOI:** 10.3390/vetsci13060565

**Published:** 2026-06-07

**Authors:** Haimei Zhou, Xianghao Xiao, Wei Lu, Yuyong He

**Affiliations:** 1 Department of Animal Science, Jiangxi Agricultural Engineering Vocational College, Zhangshu 331200, China; 18270826482@163.com; 2 Jiangxi Province Key Laboratory of Animal Nutrition and Feed, Engineering Research Center of Feed Development, Jiangxi Agricultural University, Nanchang 330045, China; xiaoxianghao10@gmail.com (X.X.); lw20030508@163.com (W.L.); 3 College of Animal Science and Technology, Jiangxi Agricultural University, Nanchang 330045, China

**Keywords:** peripheral nervous system, glial cells, fetal lungs, maternal administration, all trans retinoic acid, neonatal piglets

## Abstract

Newborn piglets in intensive pig farms are often prone to pulmonary diseases due to underdeveloped fetal lung neural systems, and ATRA is often regarded as an important morphogen that displays pleiotropic functions during embryonic development. However, information about the effect of maternal administration with ATRA on the peripheral neural system of fetal lungs is still scare. Fifteen pregnant sows were assigned to the ATRA0 (0 mg/kg diet), ATRA4, ATRA8, ATRA16 and ATRA32 treatment groups and offered their own feed from d12 to d95 after artificial insemination; then two neonatal pigs with birth weights similar to the average birth weight were taken out from each litter for the collection of lung samples, and samples were subjected to immunofluorescence staining, RNA-seq and RT-qPCR assays. Results indicated that compared to newborn piglets from the ATRA0 treatment group, newborn piglets from the ATRA4 treatment group had higher percentages of GFAP-positive astrocyte cells (*p* < 0.05) and GFAP-TUBB3 colocalization (*p* < 0.05) in the lungs. KEGG (Kyoto Encyclopedia of Genes and Genomes) analysis showed that differential expressed genes are mainly enriched in the pathways of neuroactive ligand–receptor interaction, GABAergic synapses and cell adhesion molecules. Addition of ATRA at 4 mg/kg to the diet of pregnant sows can enhance the healthy development of the pulmonary nervous systems of fetal pigs.

## Simple Summary

The peripheral nervous system has pivotal roles in regulating the health and functions of lungs, but no information has been reported about the influence of supplementing sows with ATRA (all-trans retinoic acid) during gestation on the pulmonary nervous system of fetal piglets. Data demonstrated that supplementing sows with ATRA during gestation at an appropriate concentration can improve pulmonary nervous system development by increasing the percentage of GFAP (glial fibrillary acidic protein)-positive astrocyte cells and GFAP-TUBB3 (tubulin beta 3 class III) colocalization in fetal lungs and changing the expression of genes related to neuroactive ligand–receptor interaction, GABAergic synapses and cell adhesion molecules. Addition of ATRA at 4 mg/kg to the diet of pregnant sows can enhance the healthy development of pulmonary neural tissues of fetal pigs.

## 1. Introduction

The lungs are critical organs for gas exchange and immune defense [1], and pulmonary dysfunction can lead to a series of respiratory disorders in pigs [2,3,4]. Lung function is linked to the development of the pulmonary nervous system, as the lung tissue contains a large number of neural tissues that play pivotal roles in the regulation of lung functions [5,6]. Studies have demonstrated that the pulmonary nervous system compromises a large number of neurons, glial cells, and microvessels [7]. Neurons in lung tissue primarily function in neural regulation within the lungs. They detect information such as chemical concentrations and mechanical pressures within the lungs through sensory nerve endings and deliver this information to the central system of nerves. Simultaneously, motor nerve fibers can control airway diameter by regulating the contraction and relaxation of airway smooth muscles, as well as modulate the dilation and constriction of pulmonary blood vessels and glandular secretions. These mechanisms contribute to maintaining normal pulmonary ventilation and gas exchange through reflex regulation [8,9]. Glial cells are the essential elements of the peripheral nervous system (PNS), because they have lots of functions in regulating neuronal health and function [10,11]. Glial cells in lung tissue mainly serve to support, nourish, and protect neurons [12]. Glial cells help neurons absorb nutrients and eliminate metabolic waste, and they are also involved in the metabolism and recycling of neurotransmitters, thus regulating the efficiency of nerve signal transmission. In addition, glial cells possess certain immune defense functions; when the lung tissue is damaged or infected, they can participate in the regulation of inflammatory responses, protecting neural tissue from further damage [8,10]. One of the critical functions of microvessels in the lung tissue is the gaseous interchange of oxygen and carbon dioxide between capillary walls and alveoli. This process increases the oxygen content in the blood, facilitates the expulsion of carbon dioxide from the body, maintains blood flow distribution within the lungs, and ensures efficient gas exchange [13]. Abnormal development of fetal lung neural tissue makes newborn animals prone to respiratory diseases [5,14].

In addition to satellite glia and Schwann cells, astrocyte/microglia-like cells have also been identified in the peripheral neural tissues of animals including pigs [15,16,17]. It has been reported that Schwann cells are specifically labeled with S100 [18]. GFAP is not suitable for marking SGCs (satellite glial cells) [19,20], and FABP7 (fatty acid-binding protein 7) is the most appropriate tag for SGC identification owing to its exclusive expression in the SGCs of the PNS (peripheral nervous system) [21]. Astrocyte-like cells have been identified in cardiac nexus glia and they are different from Schwann cells and satellite cells [22], and astrocytes can be recognized by the exclusively expression of GFAP [23]. Meanwhile, microglia-like cells have been identified with the microglia signature gene TMEM119 (transmembrane Protein 119) in the PNS of pigs [16], and the activated microglia are classified into homeostatic and disease-associated microglia, with TMEM119 as the signature gene for homeostatic microglia.

Currently, there are reports on the nutritional regulation of Schwann cells and satellite cells in lung tissue, but no studies have been conducted on the nutritional regulation of astrocytes and microglia in lung tissue. By examining how varying doses of ATRA supplementation in pregnant sows influence neural tissue development in fetal pig lungs, this research aims to identify an optimal ATRA supplementation dose that can reduce the occurrence of lung-related conditions in newborn piglets by promoting the healthy growth of neural tissue in the fetal pulmonary system.

## 2. Materials and Methods

### 2.1. Animal Treatment

After insemination, fifteen healthy multiparous (Second parities) crossbred sows (Landrace × Large white) with initial bodyweights of about 210 kg were randomly allotted to the ATRA0 group (0 mg ATRA/kg diet), the ATRA4 group (4 mg ATRA/kg diet), the ATRA8 group (8 mg ATRA/kg diet), the ATRA16 group (16 mg ATRA/kg diet) or the ATRA32 group (32 mg ATRA/kg diet), with 3 replicates in each group and 1 sow per replicate. Sows in each treatment group were fed twice daily (7:30 and 17:00) with the corresponding diet from d12 to d95 after insemination, and sows involved in this experiment were fed an ATRA-free basal diet at the stages of d0–d11 and d96–farrowing day [24]. The composition and nutrient level of the basal diet are listed in Table 1.

### 2.2. Collection of Lung Samples

Two healthy neonatal piglets (one male and one female) with birth weights close to the average birth weight were taken out from each litter before suckling, then bled after pentobarbital sodium treatment with a dose of 100 mg/kg birth weight using the protocol approved by the Animal Ethics Committee of Jiangxi Agricultural University (JXAULL-2024-10-01). Samples of lungs were collected from the right lobe; some of them were fixed in a 4% paraformaldehyde solution, and the remaining samples were snap-frozen with liquid nitrogen and then stored at −80 °C.

### 2.3. Immunofluorescence Staining

Samples of lungs were treated with a paraformaldehyde solution (4%) for about 12 h at 4 °C, paraffin wax-embedded, sliced into sections (5 µm thickness) and mounted on slides. After being dewaxed, dehydrated, repaired with an antigen and blocked with normal donkey serum (10%), slides were treated with the designated primary antibody (CD31-platelet endothelial cell adhesion molecule 1: Abcam PLC, Cambridge, UK, ab182981, 1:1000; GFAP: Zhejiang Oasis Biofarm Inc. Hangzhou, China, PGP055, 1:200; β3-Tubulin: Abcam, Cambridge, UK, ab18207, 1:2000; TMEM119: Oasis biofarm, Hangzhou, China, OB-PGP072-02, 1:300; PGP 9.5-protein gene product 9.5: Hangzhou Starter Biotechnology Co., Ltd., Hangzhou, China, S0B2306, 1:300) overnight at 4 °C. After washed with PBS (phosphate-buffered saline, pH 7.4), slides were treated with the designated secondary antibody (Alexa Fluor^®^594 donkey anti-rabbit lgG(H+L): Thermo Fisher Scientific Inc., Waltham, MA, USA, A21207, 1:600; Alexa Fluor^®^488 donkey anti-rabbit lgG(H+L): Thermo Fisher Scientific Inc., Waltham, MA, USA, A21205, 1:400; Donkey anti-Guinea pig IgG, AF594: Zhejiang Oasis Biofarm Inc., Hangzhou, China, D-GP594-50, 1:400) for 45 min at 37 °C, rinsed again with phosphate-buffered saline, and colored with DAPI (4′,6-diamidino-2-phenylindole, Beijing Solarbio Science & Technology Co., Ltd., Beijing, China, C0060). Stained samples were scanned with 3DHISTECH Pannoramic MIDI (3D HISTECH Ltd., Budapest, Hungary), and Case Viewer 2.4 software (3D-HISTECH Ltd., Budapest, Hungary) was used to capture target images.

### 2.4. Neural Transmitter Analysis Based on Targeted Metabolomics

Firstly, a diluent was produced by grinding the lung sample (0.02 g) using an ultrasonic cleaner (SB-5200-DTD, Ningbo Scientz Biotechnology Co., Ltd., Ningbo, China) with ultra-pure water in liquid nitrogen; homogenization was performed with 100 µL of the diluent and 400 μL of the mixture (acetonitrile/water: 8:2, *v*/*v*; mixed internal standards: 0.5 μg/mL Glycochenodexycholic acid, 0.25 μg/mL Chenodeoxycholic acid, 0.25 μg/mL Lithocholic acid, 0.25 μg/mL Ursodeoxycholic acid, and 1.0 μg/mL Cholic acid; Sanghai ZZbio Co., Ltd., Shanghai, China) by well vortexing, centrifuged in a 2 mL tube for 10 min at 4.0 °C and a speed of 12,000 rpm, and then left to stand for 30 min on ice. Secondly, the supernatant was injected into vials, and the neural transmitters in the lung samples (*n* = 6 in each group) were quantified using an UHPLC-MS/MS (ultra-high performance liquid chromatography–tandem mass spectrometry) system (ExionLC™ AD UHPLC-QTRAP^®^ 6500+, AB SCIEX Corp., Boston, MA, USA) by Novogene Co., Ltd. (Beijing, China). Thirdly, the software Analyst^TM^ 1.6.3 and MultiQuant^TM^ 3.0.2 (Sciex, Darmstadt, Germany) were used for data collection, peak picking and calibration, and RSD ≤ 15% is required for precision, accuracy and stability. Finally, an FC-value > 1.0 and *p* < 0.05 were used for the screening of distinct neural transmitters between groups.

### 2.5. RNA-Seq Analysis

Total RNA was isolated from lung samples using the TRNzol Universal Reagent kit (TianGen Biotechnology, Beijing, China). RNA-Seq libraries were constructed with the Fast RNA-seq Lib Prep Kit V2 (ABclonal, Wuhan, China). RNA sequencing was performed on the NovaSeq 6000 platform (Illumina, San Diego, CA, USA). Raw bases varied from 6 to 9 G and Q30 had a range from 94% to 97%. Raw reads were treated using fastp software (version 0.19.7), and clean reads were mapped to the reference genome (https://ftp.ncbi.nlm.nih.gov/genomes/all/GCF/000/003/025/GCF_000003025.6_Sscrofa11.1/GCF_000003025.6_Sscrofa11.1_genomic.fna.gz, https://ftp.ncbi.nlm.nih.gov/genomes/all/GCF/000/003/025/GCF_000003025.6_Sscrofa11.1/GCF_000003025.6_Sscrofa11.1_genomic.gff.gz, (accessed on 7 December 2024)) using HISAT2; the mapping rate was from 84% to 95%, and reads with mapping quality < 10 and those non-pair-mapped or multi-area-mapped were typically filtered out during gene quantification analysis. Gene expression levels were calculated as Fragments Per Kilobase of transcript per Million mapped fragments (FPKMs) using Cufflinks. DEGs (differential expressed genes) were identified with DESeq 2 using thresholds of *p* < 0.05 and |log2 Fold change (logFC)| > 2. Enrichment analysis of KEGG pathways was conducted to annotate the biological implications of DEGs [24].

### 2.6. RT-qPCR (Reverse Transcription Quantitative PCR) Assay

Total RNA was extracted from lung tissue samples using the TransZol Up Plus RNA Kit (TransGen Biotech, Beijing, China). After verifying RNA quality, reverse transcription was performed in a 20 μL reaction system consisting of 4 μL of the 5 × TransScript^®^ Uni All-in-One First-Strand cDNA Synthesis SuperMix (TransGen Biotech, Beijing, China), 1 μL of a gDNA Remover, 1 μg of the template RNA, and approximately 14 μL of RNase-free water. The reaction conditions were 5 min at 25 °C, 30 min at 42 °C, and 5 s at 80 °C. Quantitative real-time PCR (qPCR) was carried out on an ABI QuantStudio 5 instrument (Thermo Fisher Scientific Inc., Waltham, MA, USA) in a 20 μL reaction mixture containing 2 μL of cDNA, 10 μL of the 2 × Universal Blue SYBR Green qPCR Master Mix (TransGen Biotech, Beijing, China), 0.4 μL of the forward primer, 0.4 μL of the reverse primer, and around 7.2 μL of nuclease-free water. The thermal cycling conditions were an initial denaturation step at 95 °C for 30 s, followed by 40 cycles of 95 °C for 15 s (denaturation), 60 °C for 30 s (annealing), and 60 °C for 30 s (extension), with a final extension at 72 °C for 5 min. GAPDH (glyceraldehyde 3-phosphate dehydrogenase), UTS2R (urotensin 2 receptor), SLC6A1 (solute carrier family 6 member 1), GABRR1 (gamma-aminobutyric acid receptor subunit rho-1) and GABRQ (gamma-aminobutyric acid receptor, theta) were used for RT-qPCR, and the primer sequences of the genes used for RT-qPCR are provided in Table 2. GAPDH was employed as the internal reference gene. Data analysis was performed using the 2^−ΔΔCt^ method.

### 2.7. Data Treatment

The R software (version 3.6.1; R Foundation for Statistical Computing, Vienna, Austria) was run for data treatment. The differences among groups were examined using the non-parametric Kruskal–Wallis rank-sum test, and the differences between groups were determined using the non-parametric Wilcoxon test. Results are presented as the mean ± standard error of the mean (SEM), and *p* < 0.05 was set as the threshold for statistical significance.

## 3. Results

### 3.1. Microvessel Density in Lung Tissue of Neonatal Piglets Delivered by Sows from Different All-Trans Retinoic Acid Treatment Groups

**Figure 1:**
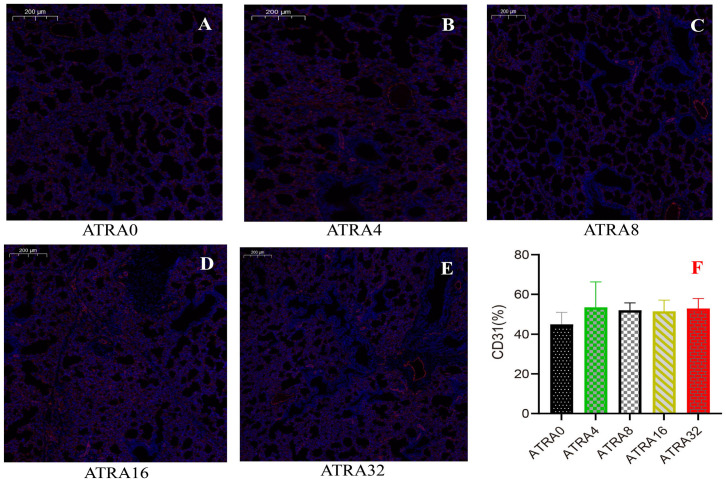
Immunofluorescence of lung sections of neonatal pigs stained with CD31 (blood vessel marker). (**A**–**E**): A, B, C, D and E are the representative images of immunofluorescence staining with CD31 for lung sections of neonatal pigs from treatment groups ATRA0, ATRA4, ATRA8, ATRA16 and ATRA32, respectively; Blue: DAPI staining, Red: CD31-positive staining; Scale bar: 200 μm; Magnification: 5×. (**F**): quantification of CD31-positive staining in lung sections from neonatal pigs across different ATRA treatment groups. All values are presented as the mean ± standard error of the mean (SEM), and statistically significant differences between groups are indicated.

Data analysis revealed that maternal administration of ATRA led to a numerical increase in pulmonary vascular density among neonatal piglets relative to the ATRA0 group (Figure 1F). However, statistical evaluation indicated no significant intergroup differences (*p* > 0.05).

### 3.2. Expression Levels of TUBB3-Positive and GFAP-Positive Cells in Lung Tissues of Neonatal Piglets Delivered by Sows from Different All-Trans Retinoic Acid Treatment Groups

**Figure 2:**
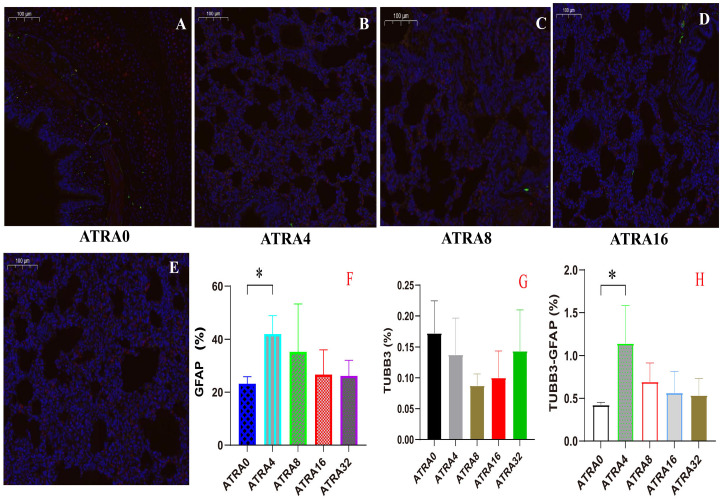
Immunofluorescence staining of lung sections from neonatal pigs marked with GFAP (astrocyte) and TUBB3 (neuron). (**A**–**E**): A, B, C, D and E are the representative images of immunofluorescence staining with GFAP and TUBB3 for lung sections of neonatal pigs from treatment groups ATRA0, ATRA4, ATRA8, ATRA16 and ATRA32, respectively; Blue: DAPI staining, Red: GFAP-positive staining, Green: TUBB3-positive staining; Scale bar: 100 μm; Magnification: 10×. (**F**–**H**): quantification of GFAP-positive staining, TUBB3-positive staining and GFAP-TUBB3-positive staining in lung sections from neonatal pigs across different ATRA treatment groups. All values are presented as the mean ± standard error of the mean (SEM), and statistically significant differences between groups are indicated; “*” indicates *p* < 0.05.

The findings revealed that maternal ATRA treatment led to an increase in the proportion of GFAP-positive astrocyte-like cells in the lungs of neonatal pigs. Specifically, neonatal pigs from the ATRA4 group exhibited a significantly higher (*p* < 0.05) percentage of GFAP-positive astrocyte-like cells in their lungs compared to those from the ATRA0 group (as shown in Figure 2F). In contrast, maternal ATRA administration showed a numerical trend towards reducing the percentage of TUBB3-positive neurons in the lungs of neonatal pigs (Figure 2G), although no statistically significant differences were observed between the groups (*p* > 0.05). Additionally, maternal ATRA treatment elevated the percentage of GFAP-TUBB3 colocalization in the lungs of neonatal pigs (Figure 2H), with neonatal pigs from the ATRA4 group displaying a higher percentage of such colocalization compared to the ATRA0 group (*p* < 0.05).

### 3.3. Expression Levels of TMEM119-Positive and PGP9.5-Positive Cells in Lung Tissue of Neonatal Piglets from Sows in Different All-Trans Retinoic Acid Treatment Groups

**Figure 3:**
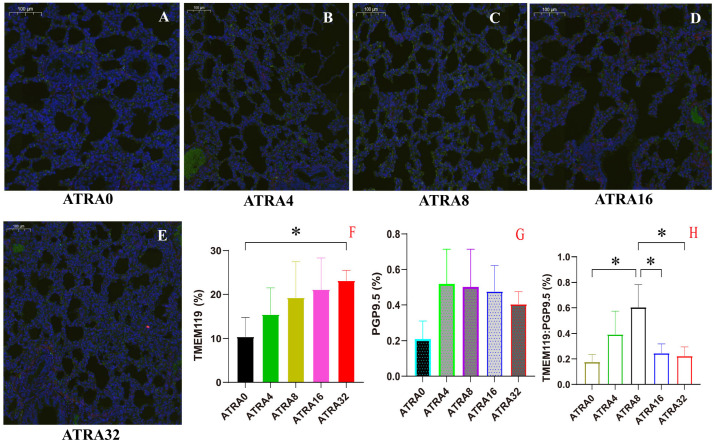
Immunofluorescence of lung sections of neonatal pigs stained with TMEM119 (microglia marker) and PGP9.5 (nerve ending marker). (**A**–**E**): A, B, C, D and E are the representative images obtained from staining of immunofluorescence targeting TMEM119 and PGP9.5 for lung sections of neonatal pigs from treatment groups ATRA0, ATRA4, ATRA8, ATRA16 and ATRA32, respectively; Blue: DAPI staining, Red: TMEM119-positive staining, Green: PGP9.5-positive staining; Scale bar: 100 μm; Magnification: 10×. (**F**–**H**): quantification of TMEM119-positive staining, PGP9.5-positive staining and TMEM119-PGP9.5-positive staining in lung sections from neonatal pigs across different ATRA treatment groups. All values are presented as the mean ± standard error of the mean (SEM), and statistically significant differences between groups are indicated; “*” indicates *p* < 0.05.

Maternal administration of ATRA led to an increase in the proportion of TMEM119-positive microglia-like cells in the lungs of neonatal pigs, as illustrated in Figure 3F. Notably, only neonatal pigs from the ATRA32 group exhibited a significantly higher percentage of these TMEM119-positive microglia-like cells in their lungs compared to those from the ATRA0 group (*p* < 0.05). When ATRA was administered to pregnant sows, the percentage of PGP9.5-positive nerve endings in the lungs of neonatal pigs showed an upward trend, as depicted in Figure 3G. However, statistical analysis revealed no significant differences in the proportion of PGP9.5-positive nerve endings among the different groups (*p* > 0.05).

Maternal ATRA treatment also exerted an influence on the colocalization percentage of TMEM119 and PGP9.5, as shown in Figure 3H. Specifically, neonatal pigs from the ATRA8 group had a significantly higher percentage of TMEM119-PGP9.5 colocalization in their lungs compared to those from the ATRA0, ATRA16, and ATRA32 groups (*p* < 0.05).

### 3.4. Concentrations of Neurotransmitters in Lung Tissue of Neonatal Piglets from Sows in Different All-Trans Retinoic Acid Treatment Groups

**Figure 4:**
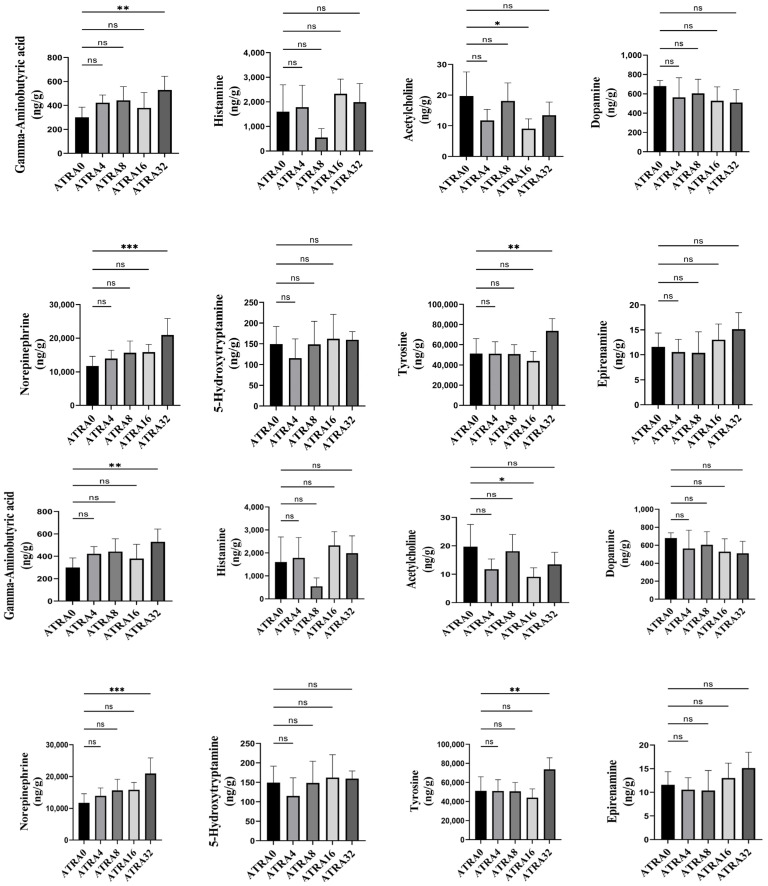
Comparison of neural transmitters in the lungs of neonatal pigs from sows fed diets with different ATRA concentrations. All values are presented as the mean ± standard error of the mean (SEM), and comparison was conducted between ATRA0 and other groups. ns, not statistically significant. *, *p*-value less than 0.05; **, *p*-value less than 0.01; ***, *p*-value less than 0.001.

As illustrated by the data presented in Figure 4, the lung tissues of neonatal pigs in the ATRA32 group exhibited significantly higher concentrations of three metabolites compared to those from the ATRA0 control group: gamma-aminobutyric acid (*p* < 0.01), norepinephrine (*p* < 0.001), and tyrosine (*p* < 0.01). When compared to the ATRA0 group, neonatal pigs in the ATRA16 group were found to have significantly reduced acetylcholine levels (*p* < 0.05) alongside a significant increase in 5-Hydroxyindoleacetate content in lung tissues (*p* < 0.01).

### 3.5. Identification and Verification of Differentially Expressed Genes That May Influence the Pulmonary Nervous System of Neonatal Piglets Born to Sows Subjected to Different ATRA Treatments

**Figure 5:**
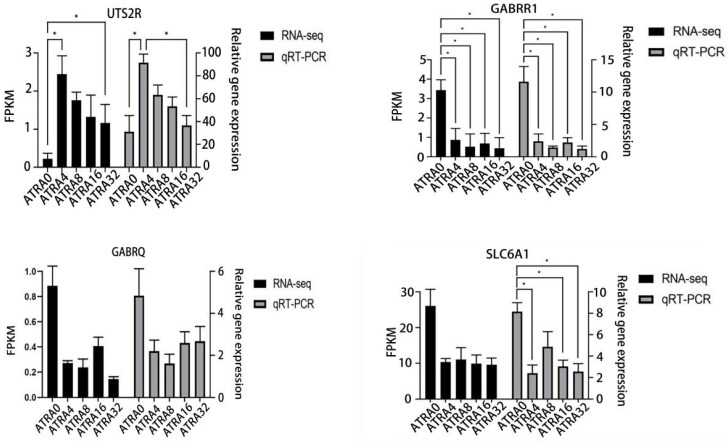
Comparison of genes expression level in lungs of neonatal pigs in different ATRA treatment groups. ATRA0, 0 mg ATRA/kg diet. ATRA0, 0 mg ATRA/kg diet. ATRA4, 4 mg ATRA/kg diet. ATRA8, 8 mg ATRA/kg diet. ATRA16, 16 mg ATRA/kg diet. ATRA32, 32 mg ATRA/kg diet. * *p* < 0.05.

Table 3 summarizes the DEGs that were either upregulated or downregulated and are hypothesized to influence the pulmonary nervous system in neonatal pigs. A striking observation was that when pregnant sows received ATRA treatment, the count of downregulated genes in the lungs of their neonatal offspring was substantially higher than the number of upregulated genes. Functional enrichment analysis revealed that these DEGs are significantly enriched in several KEGG pathways, including neuroactive ligand–receptor interaction, GABAergic synapses, primary immunodeficiency, tyrosine metabolism, mineral absorption, and cell adhesion molecules. Among these pathways, the majority of the identified DEGs were associated with the regulation of neuroactive ligand–receptor interaction and GABAergic synapses. Finally, the RT-qPCR validation results presented in Figure 5 confirmed the consistency and reliability of the transcriptome sequencing data.

## 4. Discussion

The development of fetal lungs can be regulated by ATRA, and its deficiency can lead to lung hypoplasia, agenesis, or aplasia [25]. When pregnant sows are treated with ATRA, it produces a significant positive effect on the developmental process of fetal lung tissue [24,26]. In the stage of alveolar formation, peripheral nerves are capable of releasing neurotransmitters, which in turn stimulate the proliferation, contraction and migration of alveolar myofibroblasts. This regulatory effect is essential for the normal progression of secondary septation. Meanwhile, alveolar myofibroblasts can secrete neurotrophic factors that exert reciprocal regulation on neural function. Conversely, the impairment or loss of neural activity will ultimately result in abnormal alveolar fusion and increase the thickness of the alveolar septum [27]. Our previous study demonstrated that the supplementation of 4 mg/kg of ATRA in the diet of pregnant sows effectively reduced alveolar fusion and septation thickness while increased alveolar number [24]. However, no findings reported the impact of maternal ATRA exposure on the developmental progression of neural tissue in the fetal porcine lung.

Glial cells are necessary for neuronal health and function; they actively participate in maintaining the homeostasis of the peripheral nervous system and responding to pathological insults [11]. Traditional studies suggest that glial cells in peripheral nerve tissues primarily include Schwann cells, satellite glial cells, and enteric neuroglial cells. However, some studies have also identified astrocyte-like and microglia-like glial cells within peripheral nerve tissues [15,16,17]. Astrocytes can interact with neurons and blood vessels [28], and they play roles in the following biological processes: maintaining ionic homeostasis, forming and stabilizing synapses, enabling neurovascular coupling, supporting interneuronal communication, clearing neurotransmitters, supplying energy and controlling respiratory systems [29]. A higher density of astrocytes indicates that these glial cells provide greater coverage over neurons, synapses, and blood vessels. This feature emphasizes astrocytes’ ability to regulate neuronal function, synaptic activity, and neural plasticity—an ability that is realized through their response to neurotransmitters and their regulation of blood flow [5]. Astrocytes can also facilitate vasodilation by releasing vasoactive signals via the encapsulation of vasculature with their end-feet [30]. Microglia can affect neural activity by modulating synapse formation, neural activity, and immune function [31,32].

It is reported that astrocyte lesions can result in dyspnea and hypoxemia by reducing GFAP expression and attenuating the baroreflex and chemoreflex [33]. Reduced expression of GFAP throughout embryonic development could potentially influence astrocyte maturation during key developmental time windows [34]. A study found that ATRA addition can increase the expression of GFAP and TUBB3 when glioma cells are cultured with 1 μmol/L ATRA, but it significantly decreases the expression of both GFAP and TUBB3 when cultured with 2 μmol/L ATRA [35]. The results of this study indicated that the addition of 4 mg/kg of ATRA significantly increased GFAP expression with an implication of no damages to normal baroreflex and chemoreflex. A previous study also reported that injection of hydrocortisone and budesonide into fetal sheep decreased bronchopulmonary dysplasia by significantly increasing the number of GFAP-positive astrocytes and IBA1 (Ionized calcium-binding adapter molecule 1)- positive microglia [36]. Microglia-like cells within the peripheral nervous system have pivotal roles in promoting the growth of neuronal somata and axons [16]. The results of this study also showed that ATRA addition to pregnant sows numerically increased the number of microglia in the lung tissue of newborn piglets. In addition, data obtained from the present study also demonstrated that supplementing the diet with 4 mg/kg of ATRA increased the count of co-localized astrocytes and neurons and co-localized microglia and nerve endings within lung tissues, contributing to better development of fetal lungs of pigs, and the increased colocalization of astrocytes and neurons, as well as microglia and nerve endings, can improve the development, injury recovery and function of nerves [37]. Astrocytes and microglia are also both immune cells and play crucial roles against viral neuroinfections [38], and our previous study showed that the lungs of neonatal pigs delivered by sows offered diets supplemented with 4 mg/kg ATRA had lower pathogen counts [24].

Neurotransmitters function as chemical signaling molecules that carry information across synapses, neurons and muscle cells, facilitating communication throughout the nervous system [39]. These molecules are released from the presynaptic terminals of neurons, diffuse across the synaptic cleft, and bind to specific receptors located on the postsynaptic membrane, which then triggers or suppresses physiological responses in the target receiving neuron. Glutamate serves as the main excitatory neurotransmitter in the nervous system, while GABA acts as the primary inhibitory neurotransmitter. This inhibitory function of GABA is essential for lowering the excitability of neurons and keeping a stable balance between neuronal excitation and inhibition [40]. Data of this study showed that supplementation of 4 mg/kg diet of ATRA did not significantly increase or decrease the concentration of neurotransmitters including glutamate and GABA in lung tissues of neonatal pigs.

Addition of maternal ATRA significantly affected the expression of genes associated with neural tissue development, and these differential expressed genes are primarily enriched in the pathways of neuroactive ligand–receptor interaction, GABAergic synapses, primary immunodeficiency, tyrosine metabolism, mineral absorption and cell adhesion molecules. Previous studies found that oxidative stress often causes dysfunction of neurons [41] and the UTS2R gene can protect neurons by alleviating oxidative stress [42]. The CD4 (Cluster of differentiation 4) gene has strong immuno-regulatory roles and it can protect neurons by enhancing synaptic plasticity and exhibiting anti-inflammatory effects [43]. GABA (gamma-aminobutyric acid) produces its inhibitory actions through two main classes of GABA receptors: the ionotropic types, which include GABAA (gamma-aminobutyric acid type A) and GABAC (Gama aminobutyric acid type C) receptors, and the metabotropic GABAB (Gama aminobutyric acid type B) receptors [44,45,46]. GABAA receptors aggregate at synaptic locations to maintain a high density of postsynaptic receptor populations. Upon GABA binding to these GABAA receptors, the associated ion channels undergo conformational opening, allowing chloride ions to flow either into or out of the cell [47,48]; this enables the efficient transmission of GABA-mediated inhibitory signals [49]. GABAA receptors may be categorized into multiple subtypes, which include the alpha subtype (encoded by genes GABRA1, GABRA2, GABRA3, GABRA4, GABRA5, and GABRA6), the beta subtype (encoded by GABRB1, GABRB2, and GABRB3), the gamma subtype (encoded by GABRG1, GABRG2, and GABRG3), the delta subtype (encoded by GABRD), the epsilon subtype (encoded by GABRE), the theta subtype (encoded by GABRQ), the pi subtype (encoded by GABRP) and the rho subtype (encoded by GABRR1, GABRR2, and GABRR3) [44,45]. GABAB receptors fall into the category of metabotropic receptors that couple to G proteins, and they are assembled from two distinct subunits: the GABAB receptor 1 subunit, which is commonly abbreviated as GABBR1, and the GABAB receptor 2 subunit, known as GABBR2 [50]. The downregulation of GABRR1 can reduce synaptic transmission and neurotransmitter release [51]. GABRQ and GABBR2 are involved in synaptic transmission inhibition [52]. GABRB3 acts as a crucial receptor responsible for mediating rapid inhibitory neurotransmission [53] and is a candidate gene for neurodevelopmental disorders [54]. A decrease in the expression of GABA receptors (both ionotropic and metabotropic) can weaken the inhibitory capacity of the nervous system, which can satisfy the rapid demands of oxygen for newborn piglets with faster breathing through increased neuronal excitability. Glutamate receptors are responsible for carrying out most excitatory neurotransmission processes in the nervous system and the increase in GRIK receptor level is associated with inflammatory damage and disorders of peripheral nerves [55]. Our study found that when pregnant sows were supplemented with ATRA, the expression levels of three glutamate receptor genes—GRIK1 (glutamate ionotropic receptor kainate type subunit 1), GRIK3 (glutamate ionotropic receptor kainate type subunit 3) and GRM5 (glutamate receptor metabotropic 5)—were downregulated in the lung tissue of neonatal piglets.

Neuropeptides are the principal neurotransmitters; they are involved in mucus secretion, immunoreactivity, contraction, hyperresponsiveness and inflammation during the development and progression of lung diseases [56]. NPY (Neuropeptide Y) can promote allergic airway inflammation and the Th2 response, and downregulated NPY expression can reduce the incidence of asthma [57]. KISS1 (Kisspeptin 1) plays important roles in reducing airway hyperresponsiveness of allergic asthma [58]. It is reported that TAC4 (Tachykinin 4) participates in the regulation of inflammatory processes and biological reactions to stress, and the concentration of tachykinins including tachykinin 4 is significantly elevated in the lung tissue of patients diagnosed with asthma or COPD (chronic obstructive pulmonary disease) [59,60]. PNOC (Prepronociceptin) functions in signal transduction and synaptic transmission, and asthmatic patients have high levels of PNOC [61]. Neuropeptide receptors also takes part in the function regulation of the nervous system by binding to ligands. GALR2 (Galanin receptor 2) is associated with disorders of nervous function [62]. NPFFR1 (Neuropeptide FF receptor 1) can inhibit GABAergic neurotransmission [63]. TACR1 (tachykinin receptor 1) can exacerbate inflammation [64], and in patients with inflammatory or immune-mediated neuropathy, the expression level of NPBWR1 (Neuropeptides B and W receptor 1) was significantly elevated in the peripheral nerve [65]. Elevated expression of CALCB (calcitonin gene-related peptide beta), CHRNA1 (nicotinic acetylcholine receptor subunit alpha 1), CHRNA6 (nicotine acetylcholine receptor subunit alpha 6) and CHRNA9 (nicotine acetylcholine receptor subunit alpha 9), CHRNB3 (nicotinic acetylcholine receptor beta 3) can aggravate proinflammatory cytokine production [66].

Transient receptor potential vanilloid 1, which is commonly known as TRPV1, belongs to the TRP (transient receptor potential) channel family; it can mediate the release of neuropeptides and is closely associated with pain and inflammation [67]. It has been linked to the development of both cough and asthma [68]. The results of this study indicated that ATRA administration to pregnant sows significantly downregulated TRPV1 expression. SLC6A1 and SLC6A13 function as GABA transporters [69]. SLC6A13 is a critical regulator that supports the balance between excitatory and inhibitory neurotransmission. Meanwhile, SLC6A1 mediates the transportation of GABA from the synaptic cleft into neurons and astrocytes, a process that enables the subsequent packaging and storage of GABA within presynaptic vesicles [70]. GAD1 (glutamic acid decarboxylase 1) and GAD2 are the critical enzyme for intracellular GABA synthesis, thereby influencing inhibitory neurotransmission and neural functions, and a reduction in GAD1 and GAD2 levels can decrease GABA production [46,71]. CD8A (cluster of differentiation 8 alpha) and CD8B play crucial roles in combating pathogens [72]. CD8A and CD8B are the T-cell markers. CD8B is abundantly present on T cells where this protein can form dimers with itself or CD8A to function as a cell-surface glycoprotein, and this glycoprotein can facilitate interactions between different cells and participates in the regulation of immune responses [73]. Reduced ATP1A1 expression typically leads to intracellular sodium ion accumulation and potassium ion loss, thereby disrupting membrane potential homeostasis, which may paradoxically enhance neuronal excitability [74]. SLC34A1 regulates absorption and excretion of phosphate, and excessive SLC34A1 expression results in pulmonary calcification [75]. The SLC39A4 gene can transport Zn^2+^ into cells. Zinc accumulates within the synaptic vesicles of neurons that use glutamate as their neurotransmitter [76]. Neurotoxicity in neurons and glial cells can be alleviated by decreasing zinc accumulation, because excessive zinc can generate oxidative stress [77]. Neuronal-released zinc is capable of directly triggering microglial activation, which ultimately contributes to the development of neuroinflammation [78]. This study has three primary limitations. First, the overall research workload is insufficient. Second, we were unable to isolate adequate amounts of pulmonary nerve tissue samples from the lungs of neonatal piglets to investigate the effect of maternal ATRA administration on pulmonary neural tissue. As such, we had to use whole lung tissue rather than isolated pulmonary nerve tissue to conduct immunofluorescent staining of marker genes and analyze differentially expressed genes. Third, we did not distinguish between homeostatic TMEM119-positive microglia and activated microglia.

## 5. Conclusions

Maternal ATRA treatment increases multiple key biological indicators in the lungs of neonatal piglets, including microvessel density, the proportion of GFAP-positive astrocytes, the degree of GFAP-TUBB3 colocalization, the count of TMEM119-positive microglia, the amount of PGP9.5-positive nerve endings, the level of TMEM119-PGP9.5 colocalization, as well as GABA and glutamate concentrations. Moreover, maternal ATRA exposure significantly alters the number of differentially expressed genes that participate in a series of core signaling and metabolic pathways, namely neuroactive ligand–receptor interaction, GABAergic synapses, primary immunodeficiency, tyrosine metabolism, folate biosynthesis, mineral absorption, and cell adhesion molecules. Based on the findings obtained in this research, supplementing 4 mg/kg ATRA to the diet of pregnant sows is recommended to support the development of the peripheral nervous system in the lungs of fetal pigs.

## Figures and Tables

**Table 1:** Composition and nutrient level of the basal diet (on a dry matter basis).

Composition of Basal Diet, %		Nutrient Level, %	
Corn	63.4	Digestible energy (MJ/kg)	13.10
Wheat bran	18.0	Crude protein	12.51
Beet pulp	6.4	Ether extract	3.72
Soybean meal	8.4	Crude fiber	5.26
Soybean oil	0.5	Calcium	0.78
Calcium carbonate	1.1	Total phosphorus	0.64
Calcium monophosphate	0.6	Lysine	0.55
Sodium chloride	0.5	Methionine + cysteine	0.39
Magnesium sulfate	0.1		
Premix	1.0		
Total	100		

**Table 2:** Primer information for qPCR.

Gene Name	Forward Primer (5′ to 3′)	Reverse Primer (5′ to 3′)
GAPDH	GACATCAAGAAGGTGGTGAAGCA	GTCGTACCAGGAAATGAGCTTGA
UTS2R	TCAGCGTCCCCTTAATCGTG	GTGCATGGTCAGGAAGTCCA
SLC6A1	GCAAATCTCCACGGAGGTCA	AGCGTCAGGAAGTAGGGGAT
GABRR1	GTGTTCGTGTTCCTCTCGGT	GTAGTTGCCCAGGTCGTTCA
GABRQ	AGAGGCAACTCACAAGAGCC	GGGGGACTTTCCTCATCAGC

**Table 3:** The differentially expressed genes screened from lung tissues and potentially related to the pulmonary nervous system of neonatal pigs delivered by sows supplemented with ATRA.

	ATRA4 vs. ATRA0	ATRA8 vs. ATRA0	ATRA16 vs. ATRA0	ATRA32 vs. ATRA0
Genes upregulated	UTS2R/CD4	UTS2R/ADORA3/ADRA2A	UTS2R/SELL/CTLA4	UTS2R/TSHR/PTPRC/CD4/SELL/CD4
Genes downregulated	NPY/ADORA2A/GALR3/CRHR1/TACR2/GRIK3/GHRL/UCN/GRPR/GLRA1/TACR1/KISS1/CHRNA9/GRIA1/CALCB/DRD2/NPBWR1/GHRH/SSTR3/PPYR1/GRM5/PNOC/CHRNA1/RXFP1/CRSP3/TAC4/GABRR1/GABRA5/GRIK1/GABRB3/GALR2/AVP/NPFFR1/VP/GLP1R/GABRQ/CHRNB3/GABRA6/TRPV1/CHRNA6/GABRR2/SLC6A13/GAD1/HAP1/SLC6A1/GAD2/CD8B/CD8A/ICOS/RAG1/AICDA/AOX2/ADH4/GOT1L1/MLNR/DBH/AOX4/ATP1A3/SLC39A4/SLC34A1	ADORA2A/AVPR2/DRD3/HCRT/GHSR/LYPD6B/UCN/GALR3/NPY/CHRNA9/GRIN1/NPBWR1/KISS1/PLG/PNOC/GLRA1/TACR2/GRM5/GRPR/TACR1/CHRNA1/GHRL/GRM6/GABRG3/GRM1/GHRH/AVP/DRD2/CRSP3/INSL5/CHRNA7/TAC4/NPFFR1/GLP1R/GABRB3/SSTR3/GABRQ/PPYR1/GALR2/GABRR1/GABRA5/RXFP1/GCG/GABRA6/MLNR/GRIK1/TRPV1/CHRNA6/GABRR2/SLC6A12/SLC6A13/CACNA1S/SLC6A1/GAD2/HAP1/CD8B/CD8A/AICDA/HPD/AOX2/GOT1L1/DBH/AOX4/SLC26A3/SLC34A1	GABRR2/GRM4/GLP1R/GABRR1/GLRA1/NPY/TAC4/TACR1/CHRNA6/GCG/GALR3/KISS1/GABRQ/TRPV1/GHRL/GALR2/PNOC/DRD2/UCN/CHRNA7/LYPD6B/DRD3/GRPR/TACR2/PPYR1/CRHR1/GRIK1/AVP/GRIA2/HCRT/GRIN1/GHSR/GRM6/GHRHR/GABRB3/ADORA2A/CRSP3/GABRA5/GABRA6/CALCB/GRM1/CHRNB3/CHRNA1/RXFP1/MLNR/GABRG3/SCTR/GABBR2/GRIN2C/HAP1/CACNA1A/SLC6A1/GAD2/AICDA/CD8A/ICOS/CD8B/RAG1/AOX2/AOX4/DBH/GOT1L1/ADH4/SLC34A1/ATP1A3/SLC39A4/SLC26A3	GABRR2/GLP1R/CRHR1/ADORA3/GABRR1/SSTR3/GABRQ/GLRA1/ADORA2A/GALR2/PNOC/UCN/TAC4/F2/GHRL/DRD2/GRIK1/GCG/GRPR/CHRNA7/CHRNA6/GABRB3/GABRA5/PLG/LYPD6B/GRM5/GRIN1/HCRT/GABRD/CALCB/INSL5/NPY/TRPV1/AVP/GHRH/DRD3/GRM6/GABRA6/RXFP1/CRSP3/NPSR1/CHRNB3/KISS1/TACR2/CHRNA1/NPFFR1/HRL/HAP1/SLC6A13/SLC6A1/GAD2/CACNA1S/CD8B/ICOS/MLNR/DBH/AOX2/GOT1L1/HPD/ADH4/ATP1A3/SLC34A1/SLC39A4/GALR3/SLC26A3

## Data Availability

The original contributions presented in this study are included in the article. Further inquiries can be directed to the corresponding author.
